# Piezo2 Channel Upregulation is Involved in Mechanical Allodynia in CYP-Induced Cystitis Rats

**DOI:** 10.1007/s12035-023-03386-9

**Published:** 2023-05-25

**Authors:** Lei Liu, Yan Zhao, Wenhan An, Mengmeng Zhao, Ning Ding, Hanwen Liu, Nan Ge, Jiliang Wen, Xiulin Zhang, Shulu Zu, Wendong Sun

**Affiliations:** 1 Department of Urology, The Second Hospital of Shandong University, Jinan, Shandong 250032 P. R. China; 2 Department of Radiology, Shandong Provincial Hospital Affiliated to Shandong First Medical University, Jinan, Shandong P. R. China; 3 Department of Rehabilitation, The Second Hospital of Shandong University, Jinan, Shandong P. R. China

**Keywords:** Interstitial cystitis, knock down, mechanical allodynia, primary sensory neuron, Piezo2 channel

## Abstract

Mechanical sensing Piezo2 channel in primary sensory neurons has been shown contribute to mechanical allodynia in somatic chronic pain conditions. Interstitial cystitis (IC)-associated pain is often triggered by bladder filling, a presentation that mimics the mechanical allodynia. In the present study, we aimed to examine the involvement of sensory Piezo2 channel in IC-associated mechanical allodynia using a commonly employed cyclophosphamide (CYP)-induced IC model rat. Piezo2 channels in dorsal root ganglia (DRGs) was knocked down by intrathecal injections of Piezo2 anti-sense oligodeoxynucleotides (ODNs) in CYP-induced cystitis rats, and mechanical stimulation–evoked referred bladder pain was measured in the lower abdomen overlying the bladder using von Frey filaments. Piezo2 expression at the mRNA, protein, and functional levels in DRG neurons innervating the bladder was detected by RNA-fluorescence in situ hybridization, western blotting, immunofluorescence, and Ca^2+^ imaging, respectively. We found that Piezo2 channels were expressed on most (> 90%) of the bladder primary afferents, including afferents that express CGRP, TRPV1 and stained with isolectin B4. CYP-induced cystitis was associated with Piezo2 upregulation in bladder afferent neurons at the mRNA, protein, and functional levels. Knockdown of Piezo2 expression in DRG neurons significantly suppressed mechanical stimulation–evoked referred bladder pain as well as bladder hyperactivity in CYP rats compared to CYP rats treated with mismatched ODNs. Our results suggest upregulation of Piezo2 channels is involved in the development of bladder mechanical allodynia and bladder hyperactivity in CYP-induced cystitis. Targeting Piezo2 might be an attractive therapeutic approach for IC-related bladder pain.

**Supplementary Information:**

The online version contains supplementary material available at 10.1007/s12035-023-03386-9.

## Introduction

Pain arising from the bladder is a cardinal symptom in patients with interstitial cystitis (IC) [1] that severely impairs their quality of life. A prominent feature of IC-associated pain is that it is often triggered by bladder filling (an innocuous mechanical stimulus) and is relieved after urine release. This indicates that IC-associated pain mimics “mechanical allodynia,” which is a condition in which an innocuous stimulus can induce a pain sensation whose occurrence is common in somatic chronic pain conditions. However, the underlying mechanisms for bladder chronic pain, particularly those for mechanical allodynia, in IC remain elusive [2].

In IC patients, intravesical treatment with lidocaine or botulinum toxin A reduces the pain [3, 4], which implicates a contribution of the hypersensitivity of bladder sensory afferents. The bladder primary sensory afferents consist of myelinated Aδ-fibers and unmyelinated C-fibers, which originate from cell bodies located in thoracic or lumbosacral dorsal root ganglia (DRGs). Molecular sensors, such as transient receptor potential (TRP) channels expressed in primary sensory afferents, detect mechanical and chemical changes in the bladder and convey information to the central nervous system. Sensitization of these ion channels can contribute to the hypersensitivity of bladder sensory afferents and has been implicated in chronic pain in IC patients [5] or animal models of IC [6, 7].

Piezo2 is a mechanically gated ion channel mainly expressed in DRG neurons [8]. It has received much attention for its crucial roles in the transduction of touch, vibration, and proprioception [9, 10]. However, evidence from recent studies suggests that Piezo2 might also be involved in nociception and chronic pain in inflammatory or neuropathic pain conditions [11–16]. The supporting evidence include that (1) Piezo2 has been found to be expressed in nociceptors in DRGs or trigeminal ganglions [8, 11, 14, 17]; (2) knockout of Piezo2 in mice reduces the sensitivity of mechano-nociceptors in the skin–nerve preparation [9, 14]; (3) inflammatory mediators like bradykinin or nerve growth factor (NGF) enhance Piezo2-mediated mechanosensitive currents [11] or Piezo2-mediated mechanical responses [18]; (4) inflammatory and neuropathic pain conditions are associated with elevated Piezo2 expression in DRGs [19] or trigeminal ganglia [16]; (5) knockdown of Piezo2 in DRGs inhibits inflammation-induced mechanical hyperalgesia in mouse skin [12]; and, most importantly, (6) Piezo2-knockout mice [13, 15] or humans with Piezo2 loss-of-function mutations [15] failed to develop mechanical allodynia after skin inflammation or nerve injury.

However, the above studies on the roles of Piezo2 in pain mostly focused on somatic pain, and very few studies to date have revealed the role of Piezo2 in visceral pain [20]. In particular, no study has been performed in the area of IC-associated mechanical allodynia. In this study, we aimed to investigate the contribution of the Piezo2 to bladder mechanical allodynia using a commonly employed cyclophosphamide (CYP)-induced IC model rat by investigating (1) Piezo2 expression (at the messenger RNA [mRNA], protein, and functional levels) in DRG neurons innervating the urinary bladder in control and CYP rats and (2) pain behaviors after knockdown of Piezo2 expression in DRG neurons. The role of Piezo2 in bladder overactivity in CYP rats was also examined.

## Materials and Methods

### Experimental Animals

Female Sprague–Dawley rats (2–3 months old; weight, 200–250 g) from Pengyue Animal Company (Jinan, China) were used in this study. Animals were housed in pairs or groups of four, in a 12/12 h light/dark cycle and were allowed ad libitum to feed and water. Care and handling of the animals were performed in accordance with the Shandong University Animal Care and Use Committee, and this study was approved by the ethics committee of the Second Hospital of Shandong University (KYll-2020kJA-0074).

### CYP Induced Cystitis

**Fig. 1:**
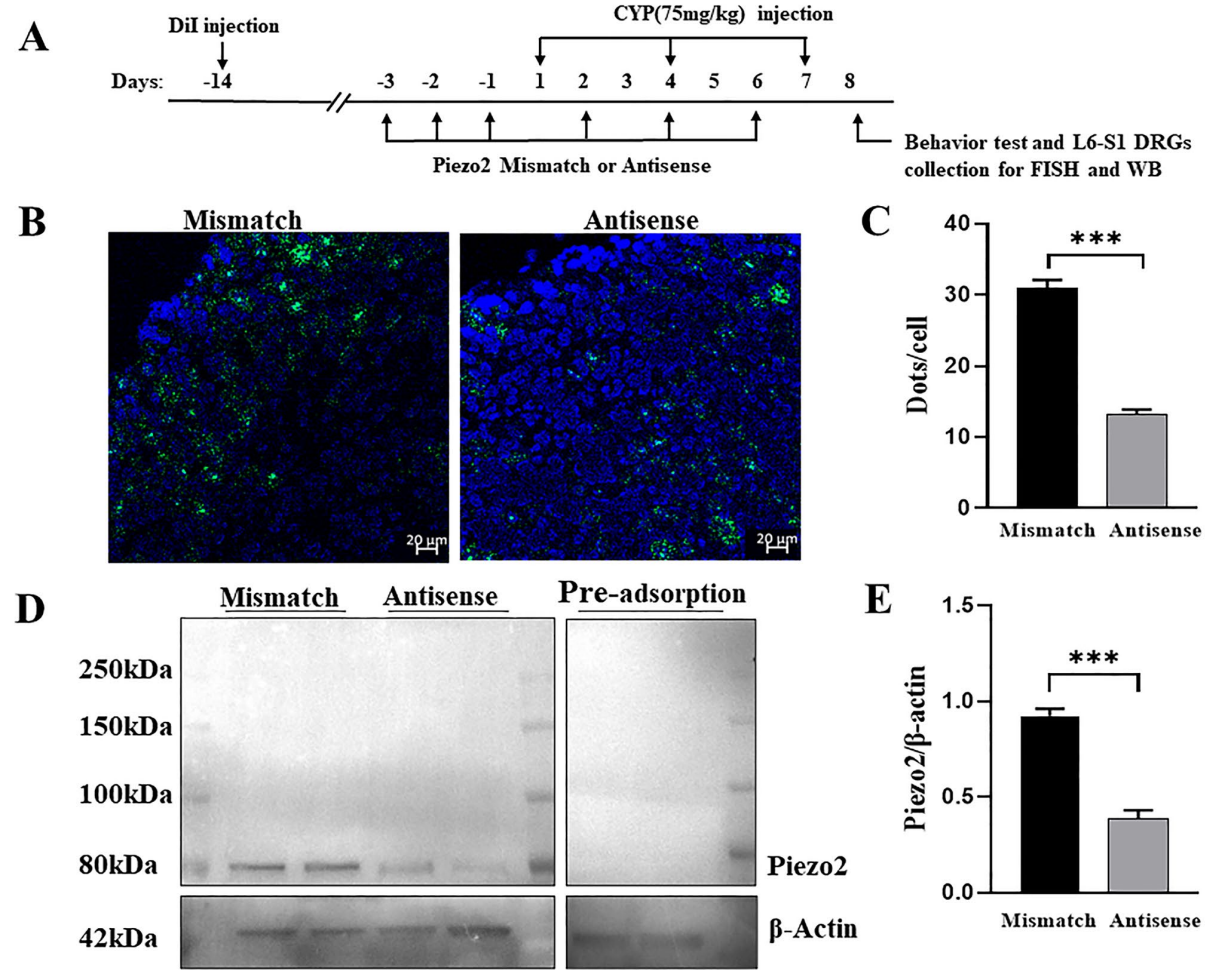
Successful knockdown of Piezo 2 in L6-S1 DRG neurons with antisense-ODNs. **(A)** The schedule for the experiments. Prior to CYP injection, Piezo2 mismatch ODNs or antisense ODNs were intrathecally injected once daily for three consecutive days and then every other day thereafter before DRG collection and behavior study. **(B-C)** FISH staining revealed that intrathecally injection of Piezo2 antisense ODNs significantly reduced Piezo2 mRNA expression in DRG neurons relative to mismatch ODNs. Summarized data from 4 rats in each group were shown in **(C)**. **(D-E)** Western blot analysis revealed that the intensity of the band at 80 kDa was significantly reduced by Piezo2 antisense ODNs relative to mismatch ODNs (**D**, left). Pre-adsorption of the Piezo2 antibody with the manufacturer’s peptide (Piezo2 antibody blocking peptide, Novus Biologicals, #NBP1-78624PEP) completely abolished Piezo2 protein bands at 80 kDa (**D**, right). Summary data **(E)** revealed a significant reduction in the ratio of Piezo2/*β*-Actin in L6-S1 DRGs of rats injected with Piezo2 antisense ODNs (*n* = 4 rats) relative to mismatch ODNs (*n* = 4 rats). Data are mean ± SEM, ***P < 0.001 (unpaired *t*-test)

Based on literature reports [21, 22], CYP (75 mg/kg; Sigma-Aldrich, St. Louis, MO, USA) was injected intraperitoneally in rats on the first, fourth, and seventh days to establish the chronic cystitis model. Rats in the control group received an equal volume of normal saline injected intraperitoneally on the same days as had been done in the CYP group. Rats were sacrificed with inhalation of overdose CO_2_ 24 h after the third CYP injection for histological examination or DRG neuron isolation (Fig. 1A).

### Knockdown Piezo2 with Anti-Sense Oligodeoxynucleotides (ODNs)

**Fig. 2:**
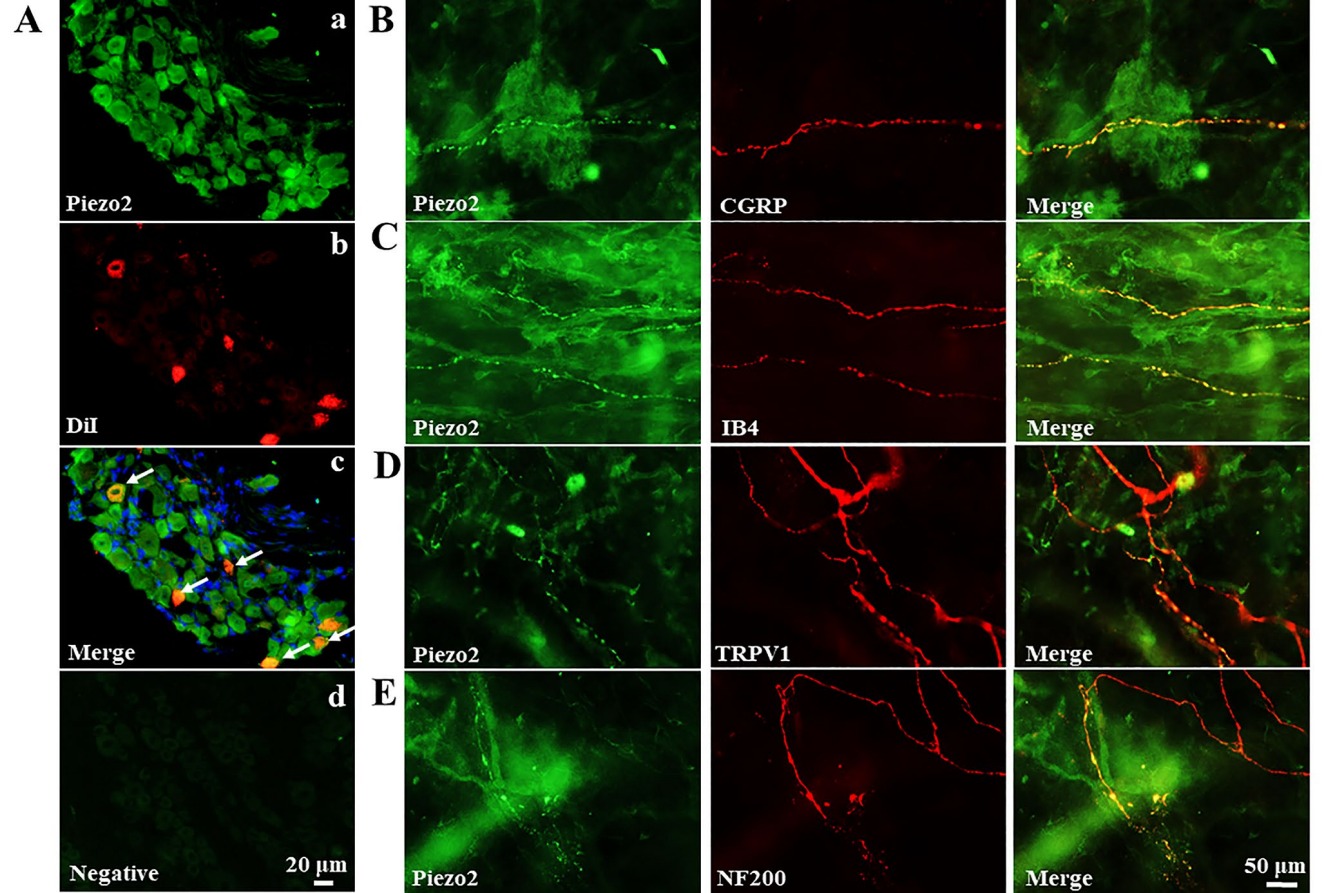
Piezo2 channels are expressed on bladder sensory afferents. **(A)** L6-S1 DRG neuron cell bodies innervating the bladder were labeled with DiI (red, **b**). Piezo2 expressing neurons was revealed as green (**a**), and their co-expression was shown in yellow (**c**, white arrows). Pre-adsorption of with the Piezo2 antibody synthetic blocking peptide (1:200, BLP-PC090; Alomone Labs, Jerusalem, Israel) completely abolishes Piezo2 staining and used as negative control (**d**). The nucleus was stained with DAPI (blue). Scale bar: 20 μm. (**B-D**) Whole mount immunofluorescence of bladder mucosa showed the co-expression of Piezo2 with CGRP (**B**), IB4 (**C**), TRPV1 (**D**) and NF200 (**E**). Scale: 50 μm

**Fig. 3:**
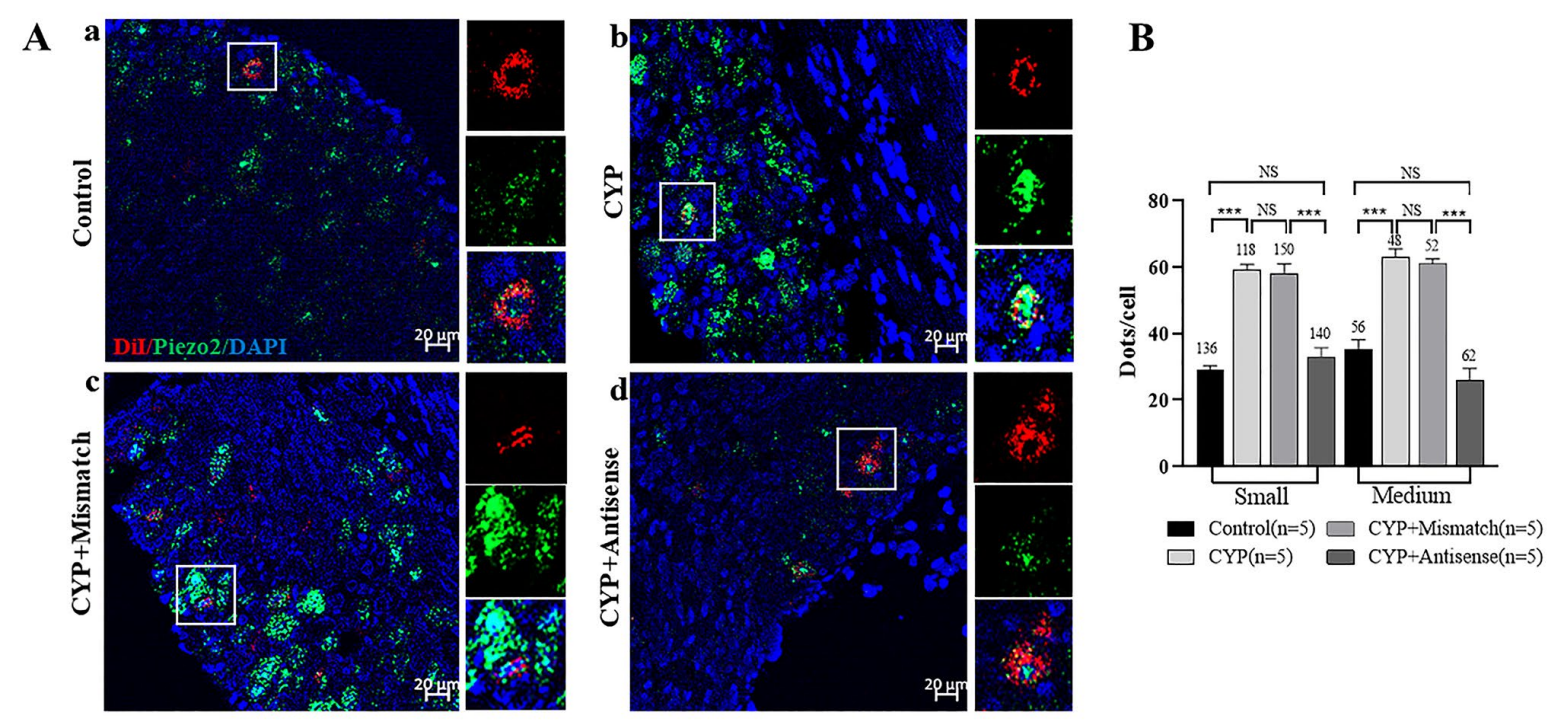
The Piezo2 mRNA upregulation in L6-S1 DRG neurons in CYP rats. (**A**) Representative fluorescent images of Piezo2 in L6-S1 DRG neurons from control (**a**), CYP (**b**), CYP with mismatch ODNs (**c**) and CYP with antisense ODNs (**d**) rats. RNAscope staining of Piezo2 mRNA is shown in green. DRG neurons innervating the bladder were labeled with DiI and are shown in red. The nucleus was stained with DAPI (blue). A representative DiI^+^ neuron in each section is magnified in the boxed areas. (**B**) Comparisons of the number of RNAscope dots per neuron for Piezo2 between four group of rats in small and medium sized DiI^+^ neurons. The number of RNAscope dots for Piezo2 was significantly increased in both small and medium sized DiI^+^ neurons from CYP and CYP + mismatch ODNs rats compared to control rats (two-way ANOVA, p < 0.001). However, the number of dots were significantly reduced in DRG neurons from CYP + antisense ODNs rats. The number above each bar indicates the number of neurons. Values are presented as mean ± SEM. n is the number of rats for each group. Scale bar = 20 μm. NS: no significance. ***P<0.001

**Fig. 4:**
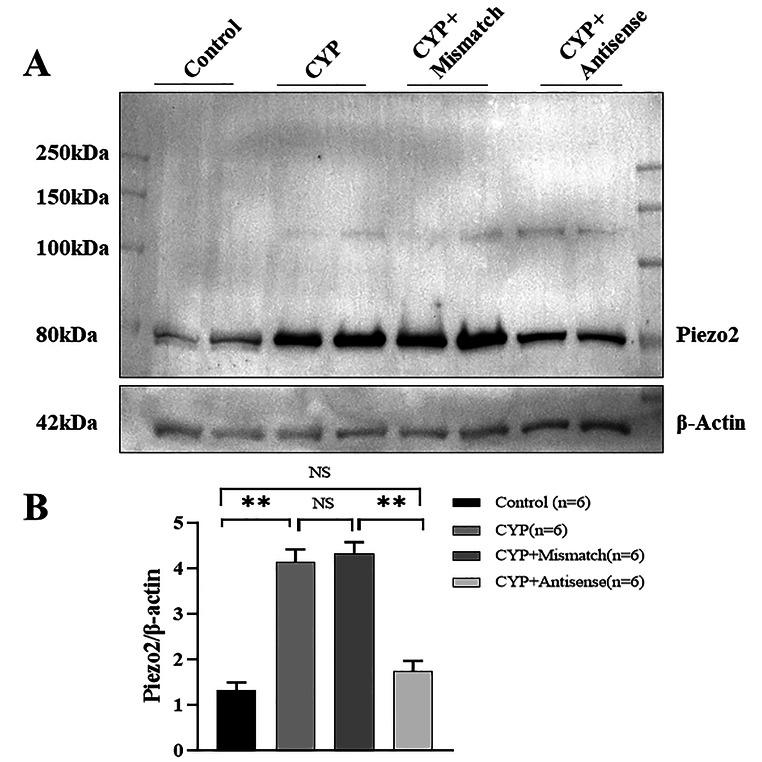
Upregulation of Piezo2 protein expression in L6-S1 DRG neurons in CYP rats. (**A**) Representative western blots of protein expression of Piezo2 channels. Total membrane proteins were extracted from bilateral L6-S1 DRGs from four group of rats. β-actin (42 kDa) was used as the control. (**B**) Summary western blot data showing upregulated protein expression of Piezo2 in L6-S1 DRGs in CYP rats; and this increase was significantly reduced in antisense + CYP rat (two-way ANOVA, p < 0.01). Data are presented as mean ± SEM. n is the number of rats in each group. ** p < 0.01

Because no specific agonist and antagonist were available for the Piezo2 channel, in our study, the role of Piezo2 in bladder pain was tested using a Piezo2-knockdown strategy. We referred to Nencini et al.’s study [18] to knock down Piezo2 in DRGs with anti-sense ODNs. The following 3 ODNs to rat Piezo2 mRNA were applied: 5’-CCACCACATAAACACCTGC-3’, 5’-TTCCTCCTCTTCACTATCCG-3’, and 5’-CCTCAATGGTTTCCGTAGTTC-3’ (Genepharma, Shanghai, China). For control experiments, the following mismatched ODNs were used: 5’-ACATCACACGAACTCCAGC-3’, 5’-GTCATCGTCATCACATTGCG-3’, and 5’-TCTCAGTGCTCTCCATAGGTA-3.’ To enable them to have a longer action, the anti-sense ODNs were modified by 2’-OMe and 5’-Chol. In brief, under isoflurane anesthesia, the anti-sense or mismatched ODNs (3.5 µg /µL, in a volume of 20 µL) were injected into the lumbar subarachnoid space between the L4 and L5 vertebra with a Hamilton syringe. Injections were conducted once daily on 3 consecutive days before CYP injection and then every other day thereafter before DRG collection and behavior study (Fig. 1A, 2, 3,4).

Successful Piezo2 mRNA knockdown in the L6–S1 DRGs in naïve rats was confirmed by FISH experiments compared to the injection of the mismatched ODNs (Fig. 1B and C). Western blot analysis revealed a significant reduction in the density of Piezo2 protein at 80 kDa band (Fig. 1D and E) in DRGs from knockdown rats.

### DRG Neuron Labeling

DRG neurons innervating the urinary bladder were labeled by retrograde axonal transport of 1,1’-dioctadecyl-3,3,3’,3’-tetramethylindocarbocyanine perchlorate (DiI) (Invitrogen, Carlsbad CA, USA). Ten to fourteen days prior to immunofluorescence and Ca^2+^ imaging (Fig. 1A), 17 mg/mL of DiI in saline diluted from a stock of 170 mg/mL in dimethyl sulfoxide was injected into the bladder wall (5 sites at 2 µL/site) under isoflurane anesthesia with a 30-g needle. The needle was kept in place for 20 s, and any leakage of dye was removed by a cotton swab. The injection site was further washed with saline to minimize contamination of adjacent organs. The abdominal muscles and overlying skin were sutured after injection and rats were allowed to recover for at least 4 days. DiI-labeled DRG neurons were easily identified under epifluorescence illumination with a Texas-red/rhodamine filter set. Cells were considered as DiI^+^ if the mean fluorescence exceeded five times the standard deviation of the background fluorescence.

### Whole-Mount Bladder Nerve and DRG Immunofluorescence Staining

*Whole-mount bladder nerve staining* Rat bladder was removed under isoflurane anesthesia, and the mucosa was dissected from the smooth muscle layer with a fine forceps. The mucosa was fixed in 4% paraformaldehyde for 2 h. Then, mucosa was incubated in 1 mL of blocking buffer (containing 1% Triton-X100, 2% bovine serum albumin [BSA], and 4% normal goat serum in phosphate-buffered saline) for 4 h. The tissues were then incubated with rabbit anti-Piezo2 (diluted at 1:100 in phosphate-buffered saline, APC090; Alomone Labs, Jerusalem, Israel) and neurofilament 200 (NF200) (1:200; #2836; Cell Signaling Technology, United States) or mouse anti-calcitonin gene-related peptide (anti-CGRP) (1:100, ab81887; Abcam, Cambridge, UK), TRPV1 (1:100, 66983-1-Ig; Proteintech, China), or isolectin B4 (IB4) (diluted at 10 µg/mL in phosphate-buffered saline, DL-1207; Vector Laboratories, Burlingame, CA, USA) for almost 40 h at 4 °C. Then, the sections were incubated with the secondary antibodies (Elabscience Biotechnology Co., Ltd., Wuhan, China) Alexa Fluor 594–conjugated goat anti-mouse immunoglobulin G (H + L, 1:200) or fluorescein-conjugated goat anti-rabbit immunoglobulin G (H + L, 1:50) overnight at 4 °C.

*DRG staining* A laminectomy was performed under urethane anesthesia, and L6-S1 DRGs were removed and fixed in 4% paraformaldehyde. Paraffin sections of DRGs (5 μm) were incubated with the Piezo2 antibody (APC090; Alomone Labs, Jerusalem, Israel) overnight at 4 °C, while other procedures used for DRG immunofluorescence staining were the same as described in whole mount nerve section.

The staining results were analyzed using a confocal laser scanning microscope (ZEISS Observer.Z1; Carl Zeiss Microscopy GmbH, Jena, Germany). Positive staining was considered if the mean fluorescence exceeded five times the standard deviation of the background fluorescence. For whole mount staining, 4 pieces of mucosa per rat were stained and visualized. Piezo2 positive DRG neurons were counted on 2–3 sections of each DRG, and L6-S1 DRGs from 4 rats were analyzed.

### Mechanical Sensitivity Testing

As previously reported [21, 23], we measured rat withdrawal behaviors in response to mechanical stimulation in the lower abdominal region overlying the bladder as a substitute for referred bladder pain in CYP rats. Each rat was placed in a poly (methyl methacrylate) chamber (6 × 10 × 12 cm) for acclimatization for ~ 10 min prior to testing. Then, von Frey filaments with forces of 0.04, 0.07, 0.16, 0.4, 1.0, 1.4, and 2.0 g were applied to the pelvic region overlying the bladder. Each filament was applied 10 times for ~ 1 s with an inter-stimulus interval of 2–5 s in ascending order of force. The following 3 types of withdrawal behaviors were considered to be positive responses: (1) sharp retraction of the abdomen, (2) immediate licking or scratching of the stimulation area, and (3) jumping. For each force, the response frequency in response to the 10 stimulations was determined.

### Fluorescence in Situ Hybridization (FISH)

FISH was performed on paraffin-embedded sections of bilateral L6–S1 DRGs (5 μm), as described previously [22]. In brief, RNA transcripts were detected with the RNAscope™ version 2.0 assay (Advanced Cell Diagnostics, Newark, CA, USA) using an RNAscope™ fluorescent multiplex reagent kit (#320,850) and probes for Piezo2 (REF:549,741, ACD). All the DRG sections have be processed in parallel under identical conditions. The slides were viewed using confocal laser scanning microscopy, and images were acquired using ZEN 2.1 (blue version; Carl Zeiss Microscopy GmbH). The Qupath-0.2.3 software was used for the analysis. Individual RNAscope™ dots in the green channel were detected using a rolling-ball filter (1 μm). Cells containing ≥ 5 RNAscope™ dots were considered positive expression. 2–3 sections were visualized for each DRG, and at least 4 nonoverlapping fields for each section were examined at 20X. L6-S1 DRGs from 5 rats in each group were analyzed.

### Western Blotting

Western blotting was performed as described previously [22]. Briefly, proteins were extracted from L6–S1 DRGs, separated by sodium dodecyl sulfate–polyacrylamide gel electrophoresis, and transferred onto polyvinylidene fluoride membranes. After blocking with 5% skim milk, the membranes were incubated overnight at 4 °C with Piezo2 rabbit polyclonal primary antibodies (1:1000, NBP1-78624; Novus Biologicals, Littleton, CO, USA), then with horseradish peroxidase–conjugated secondary antibodies. Protein bands were detected using an enhanced chemiluminescence kit. The band density was quantified using a computer-assisted imaging analysis system (ImageJ; U.S. Institutes of Health, Bethesda, MD, USA).

### Piezo2 Antibody Specificity

The Piezo2 antibody used in western blot experiments (Figs. 1D and 4A) is the polyclonal rabbit anti-Piezo2 from Novus Biologicals (#NBP1-78624; RRID: AB_11005294). The ability of this antibody to detect Piezo2 has been confirmed by antibody-mediated affinity purification of native Piezo2 from mouse DRG, followed by mass spectrometry and label-free quantification [24]. The specificity has also been verified with Piezo2cKO mice [25] and has been confirmed in many literature reports [18, 26, 27]. In line with previous reports [24, 26, 27], with this antibody our Western blot analysis reveals the 80 kDa band in DRG neurons (Fig. 1D). However, we did not detect the band at *>* 250 kDa [18]. In the current study, we have further shown that pre-adsorption of the Piezo2 antibody blocking peptide (Novus Biologicals, #NBP1-78624PEP) completely abolishes bands at 80 kDa in rat DRG (Fig. 1D), and that the intensity of the band are significantly reduced by antisense treatment targeted specifically at Piezo2 (Fig. 4A). Somehow, with this Piezo2 antibody we could not detect signals in DRG neuron or bladder in our immunofluorescence experiments.

The Piezo2 antibody used in immunofluorescence (Fig. 2) was obtained from Alomone (APC090, Jerusalem, Israel). It is also a rabbit polyclonal antibody which is raised and affinity purified on immobilized antigenic peptide (RTIFHDITRLHLD, 12/13 homologous to the rat sequence) corresponding to 13 amino acid residues (1092–1104) of human Piezo2. Manufacturer’s in-house data show that this antibody detects Piezo2 expression in rat DRG lysates and signals are blocked with antigenic peptide preabsorption. The specificity of this Piezo2 antibody in immunofluorescence of rat DRG and trigeminal ganglions has been confirmed with several strategies [19]. To further test Piezo2 antibody specificity, in the present study DRG sections were co-incubated with the synthetic blocking peptide (1:200, BLP-PC090; Alomone Labs, Jerusalem, Israel) (Fig. 2Ad).

### DRG Neuron Culture

After a laminectomy under urethane anesthesia, L6–S1 DRGs were removed and rats were euthanatized with inhalation of overdose CO_2_. Ganglia were digested for 30 min at 37 °C in minimum essential medium (Gibco Laboratories, Gaithersburg, MD, USA) containing collagenase 4 (2 mg/mL) and trypsin (1 mg/mL; Worthington Biochemical, Lakewood, NJ, USA) and mechanically dissociated. The digestion was stopped by adding 10% FBS, and the cell suspension was centrifuged (5 min at 1000 rpm). Cells were re-suspended in serum-free Dulbecco’s modified Eagle medium and plated on poly-L-lysine–coated (Sigma-Aldrich) glass coverslips. Cells were incubated at 37 °C in 95% O_2_/5% CO_2_ and 90% humidity for 2–4 h before Ca^2+^ imaging.

### Ca^2+^ Imaging and Single-Cell Mechanical Stimulation

Ca^2+^ imaging was performed as described in our previous study [22]. Briefly, DRG neurons were loaded with Fura 2-AM (2 µM; Dojindo Laboratories, Tongren, Japan) for 30 min at 37 °C. Fura 2-AM was dissolved in Hank’s balanced salt solution containing 138 mM of NaCl, 5 mM of KCl, 0.3 mM of KH_2_PO_4_, 4 mM of NaHCO_3_, 2 mM of CaCl_2_, 1 mM of MgCl_2_, 10 mM of HEPES, and 5.6 mM of glucose, with a pH of 7.4. Cells were excited alternatively at 340 and 380 nm, and the fluorescence emission was detected at 510 nm using a computer-controlled monochromator. Image pairs were acquired every 1–30 s, and the acquisition of images were controlled using a dynamic image analysis system (MetaFluor® imaging software; Molecular Devices, San Jose, CA, USA). The ratio of the fluorescence signal measured at 340 nm divided by the fluorescence signal measured at 380 nm was used to measure the increase in [Ca^2+^]_i_. A significant increase in [Ca^2+^]_i_ was considered if the change in peak ratio of 340 /380 *>* 0.1.

We referred to our previous study [28] for single-cell mechanical stimulation. Briefly, a motorized MP-285 micromanipulator (Sutter Instruments, Novato, CA, USA) was used for controlling glass micropipette movement. DRG neurons were mechanically stimulated by deflection of the plasma membrane using a glass micropipette with a fine, closed, and rounded tip. The micropipette was lowered in steps of 3 μm to induce membrane deflection. Mechanical stimulus evoked peak increase in [Ca^2+^]_i_ was measured with Ca^2+^ imaging.

### Voiding Behavior Measurement

*Cystometrogram (CMG) recording* CMGs were performed as described previously [22]. Briefly, a midline abdominal incision was made under urethane anesthesia and a PE-50 tube was inserted into the bladder through the dome. Then, saline solution (37 °C) was infused at 0.04 mL/min and the bladder contractions were measured with a pressure transducer connected to a data-acquisition system (AD Instruments Pty. Ltd., New South Wales, Australia). The inter-contraction interval (i.e., the time between 2 reflex bladder contractions) and the pressure threshold for voiding initiation were recorded.

*Urine spot assay* The procedures used are referred to in our previous study [22]. Rats were placed individually in polycarbonate cages with 46- × 57-cm filter papers (catalog no. 2300 − 917; Whatman, Maidstone, UK) taped to the floor. Rats were left in a darkened room for 4 h without access to water. Then, filter papers were viewed under incident ultraviolet light (AutoChemi bioimaging system; UVP, Upland, CA, USA) to reveal urine spots. Images of the papers were electronically captured. Video recordings were also performed during urine spot assay to determine the overlapping voids.

### Statistical Analysis

Data are reported as mean ± standard error of the mean (SEM) values. Statistical analysis was performed using an unpaired 2-tailed Student’s *t* test for comparisons between 2 groups and a 1‐ or 2‐way analysis of variance followed by the Holm-Sidak test for comparisons of multiple groups (GraphPad Prism version 8.00; GraphPad Software, San Diego, CA, USA). *P* < 0.05 was considered to be statistically significant.

## Results

### Piezo2 Channels are Expressed in Bladder Primary Sensory Afferents

To reveal the potential role of the Piezo2 channel in bladder nociception and pain, we first investigated Piezo2 expression in bladder primary sensory afferents with immunofluorescence. L6–S1 DRG neurons innervating the bladder were labeled with the tracer DiI (red, Fig. 2Ab). DiI^+^ neurons from L6–S1 DRG neurons have diameters ranged from 20 to 35 μm. Immunofluorescence staining demonstrated that most of the DiI^+^ neurons (288/306 neurons from 4 rats, 94.1%) expressed Piezo2 (Fig. 2Aa and 2Ac). Among them, 199 of 213 (93.4%) small-sized (< 30 μm) and 89 of 93 (95.7%) medium-sized (≥ 30 μm) DRG neurons expressed Piezo2, respectively. To note, most of the DiI^−^ neurons (1467/1502 neurons, 97.7%) also expressed Piezo2 (Fig. 2Aa), indicating a pan-expression of Piezo2 in L6–S1 DRG neurons. DRG sections lost piezo2 staining after treatment with synthetic piezo2 antibody blocking peptide (Fig. 2Ad), which confirmed the specificity of piezo2 antibody.

Next, we investigated Piezo2 expression in bladder sensory nerve terminals with whole-mount nerve immunofluorescence staining of the bladder mucosa. Nerve terminals were stained with NF200 (a marker of myelinated nerves), CGRP (a marker of peptidergic C-fibers), IB4 (a marker of non-peptidergic C-fibers), and TRPV1 (a marker of nociceptive fibers). As demonstrated in Fig. 2B–E, Piezo2 channels were expressed in CGRP^+^, IB4^+^, TRPV1^+^ and NF200^+^sensory nerves in the bladder mucosa.

### Cystitis is Associated with Piezo2 mRNA Upregulation in Bladder Afferent Neurons

To investigate the involvement of Piezo2 in cystitis-associated pain, Piezo2 mRNA expression in DRG neurons innervating the bladder was examined using the FISH approach. We classified DRG neurons into small (diameter < 30.0 μm) and medium (diameter ≥ 30 μm) sizes, which are thought to project C- and Aδ- fibers, respectively [29]. Be consistent with our immunofluorescence findings (Fig. 2), Piezo2 mRNA was found in most of the DRG neurons from control rats (1456/1598, 91%, Fig. 3Aa). The Piezo2 mRNA in both small- (61.0 ± 1.5 vs. 29.1 ± 1.3 dots/cell, *P* < 0.001) and medium-sized (63.2 ± 2.5 vs. 35.3 ± 3.1 dots/cell, *P* < 0.001) DiI^+^ DRG neurons were increased in CYP rats (Fig. 3A and B). Of note, Piezo2 mRNA was also increased in both small- and medium-sized DiI^−^ DRG neurons in CYP rats (57.3 ± 1.7 vs. 24.2 ± 2.5 dots/cell for small-sized neurons, *P* < 0.001; 60.7 ± 1.4 vs. 23.4 ± 2.1 dots/cell for medium-sized neurons, *P* < 0.001). In CYP rats, successful Piezo2 mRNA knockdown in L6–S1 DRGs with its ODNs was revealed compared to with the mismatched ODNs (Fig. 3Ac vs. 3Ad).

### Cystitis is Associated with Piezo2 Protein Upregulation in L6–S1 DRG Neurons

Western blotting was conducted to evaluate the protein expression of Piezo2 channels in L6–S1 DRGs. Compared to control rats, CYP rats showed a 3.1-fold increase in Piezo2 protein (Fig. 4A, B and P < 0.01). Compared to the injection of mismatched ODNs, knockdown of Piezo2 mRNA significantly decreased Piezo2 protein expression in DRGs from CYP rats (Fig. 4A, B and P < 0.01).

### Cystitis is Associated with Functional Piezo2 Upregulation in Bladder Afferent Neurons

**Fig. 5:**
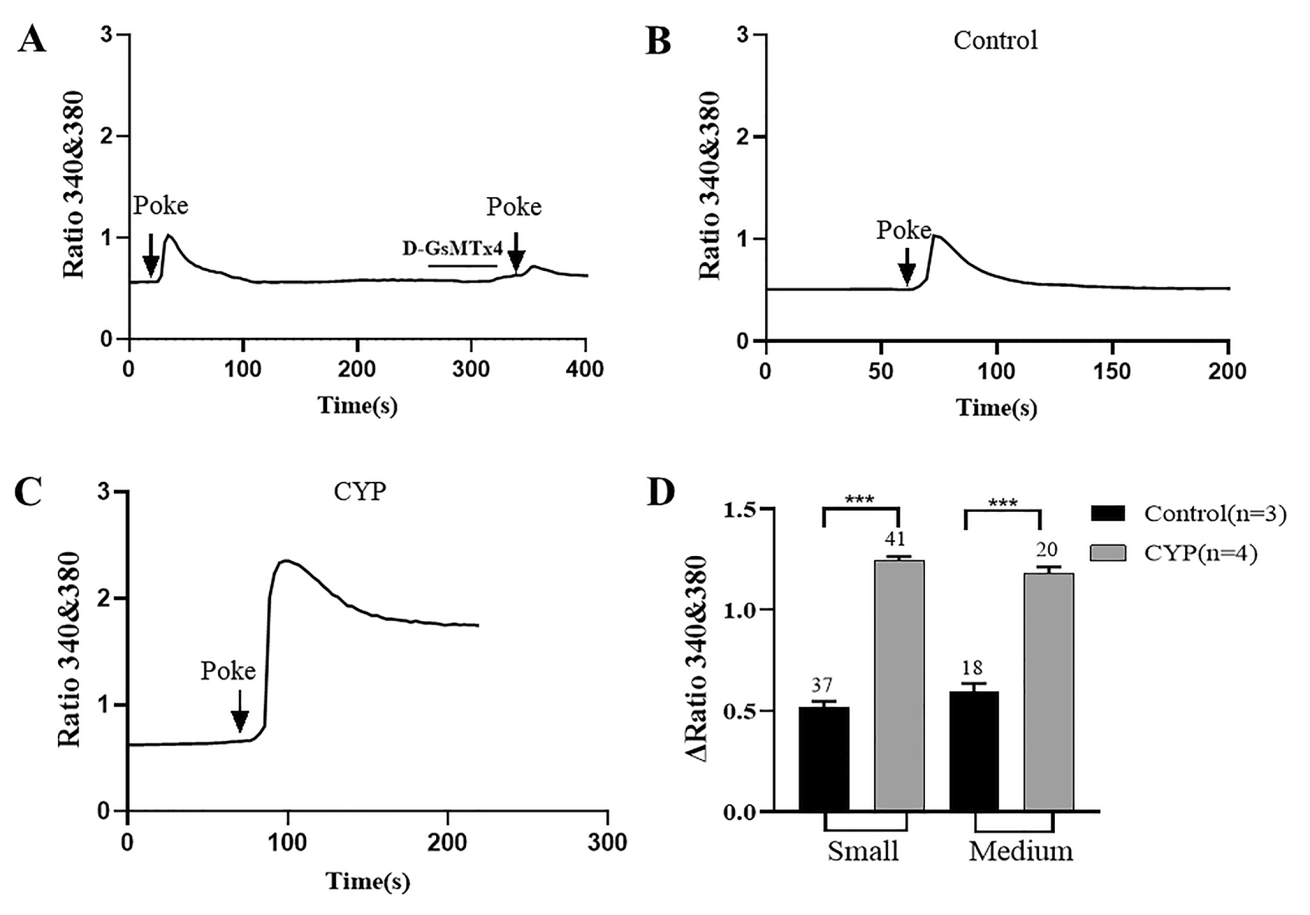
Piezo2 activity is enhanced in L6-S1 DRG neurons from CYP rats. (**A**) Representative trace showing mechanical stimulation (3 μm membrane deflection) induced intracellular Ca^2+^ increase was reduced with pretreatment of D-GsMTx4 (5µM), an Piezo2 channel antagonist. (**B-C**) Representative traces showing intracellular Ca^2+^ increases induced by a mechanical stimulation in DiI^+^ DRG neuron from control (**B**) and CYP (**C**) rats. (**D**) Comparisons of the peak amplitude of the mechanical stimulation evoked Ca^2+^ increase between control and CYP rats. In both small and medium sized DiI^+^ neurons, there was a significant increase in peak amplitude of the Ca^2+^ increase in CYP rats than control rats (unpaired *t*-test). Values are presented as mean ± SEM. The number above each bar indicates the number of neurons. n indicates the number of rats. ***P<0.001

To examine the changes in functional Piezo2 expression in CYP rats, the [Ca^2+^]_i_ increase induced by a mechanical stimulus in DRG neurons was compared between control (n = 3) and CYP (n = 4) rats. Mechanical stimulation (a 3-µm membrane deflection) was provided by poking with a glass micropipette, and the evoked peak [Ca^2+^]_i_ increase was significantly reduced in the presence of D-GsMTx4 (5 µM, Tocris, Minneapolis, MN, USA), an Piezo2 antagonist [30, 31] (Fig. 5A), indicating that the Piezo2 channel mediates the response to mechanical stimulation. The peak amplitude of the mechanical stimulus–induced [Ca^2+^]_i_ increase was significantly greater in both small- (1.23 ± 0.05 vs. 0.52 ± 0.07, *P* < 0.001) and medium-sized (1.18 ± 0.08 vs. 0.60 ± 0.10, *P* < 0.001) DiI^+^ DRG neurons from CYP rats compared to control rats (Fig. 5B-D).

### Knockdown of Piezo2 expression in DRG neurons attenuated mechanical stimulus–evoked referred bladder pain in CYP rats

**Fig. 6:**
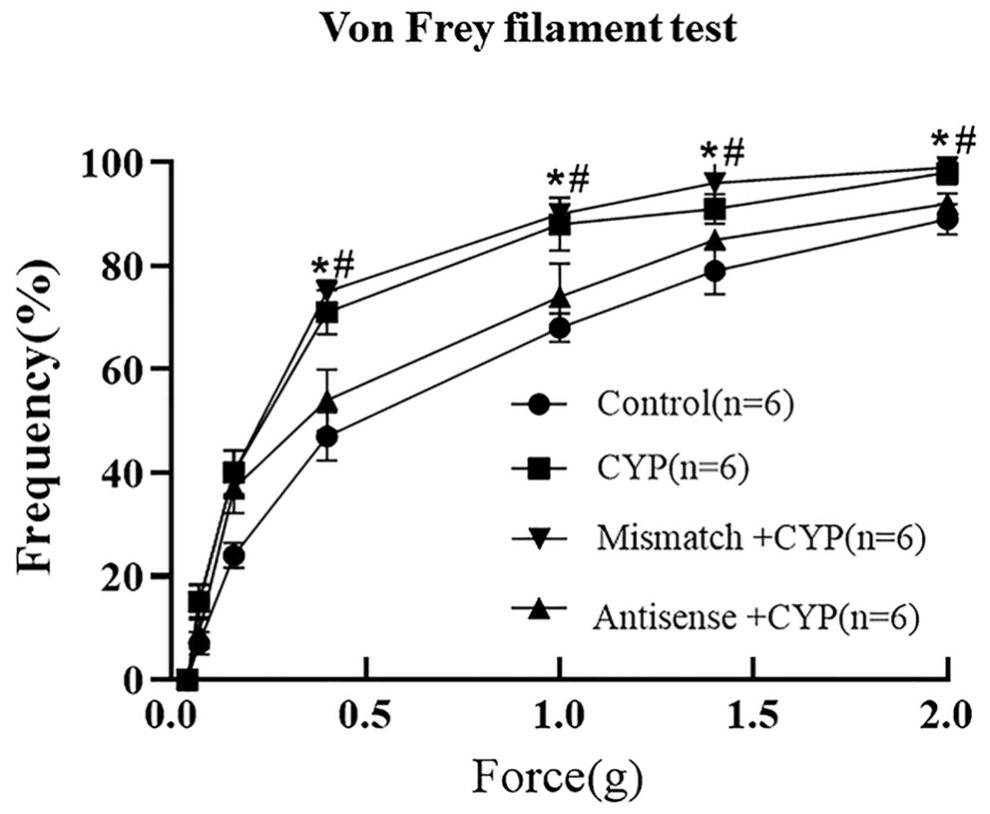
Knockdown Piezo2 expression in sensory neurons attenuated mechanical allodynia in CYP rats. Mechanical allodynia in lower abdominal region was assessed using a series of von Frey filaments (0.04-2 g) on the day after the third CYP injection. CYP rats and CYP + mismatch rats showed an increased response to mechanical stimulation (> 0.4 g) compared with control rats, and these increased responses were significantly reduced in antisence + CYP rats (Two-way repeated measure ANOVA, p < 0.05). *p < 0.05 compared with control, #p < 0.05 compared with CYP + Mismatch. n indicates the number of rats

To determine whether increased Piezo2 expression in DRG neurons contributed to the bladder pain in CYP rats, the mechanical stimulus–evoked pain behaviors were compared between control and CYP rats and between Piezo2 mismatched ODN–treated CYP (mismatched + CYP) rats and anti-sense ODN–treated CYP (anti-sense + CYP) rats. We measured rat withdrawal behaviors in response to Von Frey filaments (0.04–2.0 g) stimulation in the lower abdominal region overlying the bladder as a substitute for referred bladder pain in CYP rats. As expected, increased pain responses were detected in the CYP and mismatched + CYP rats compared to control rats. However, pain behaviors were significantly reduced in the anti-sense + CYP rats (Fig. 6). To note, knocking down piezo2 expression in naïve rats also reduced mechanical stimulation (0.4-2.0 g) evoked responses by 8–11% (n = 4 rats, data not shown).

### Knockdown of Piezo2 Expression Attenuated Bladder Overactivity in CYP Rats

**Fig. 7:**
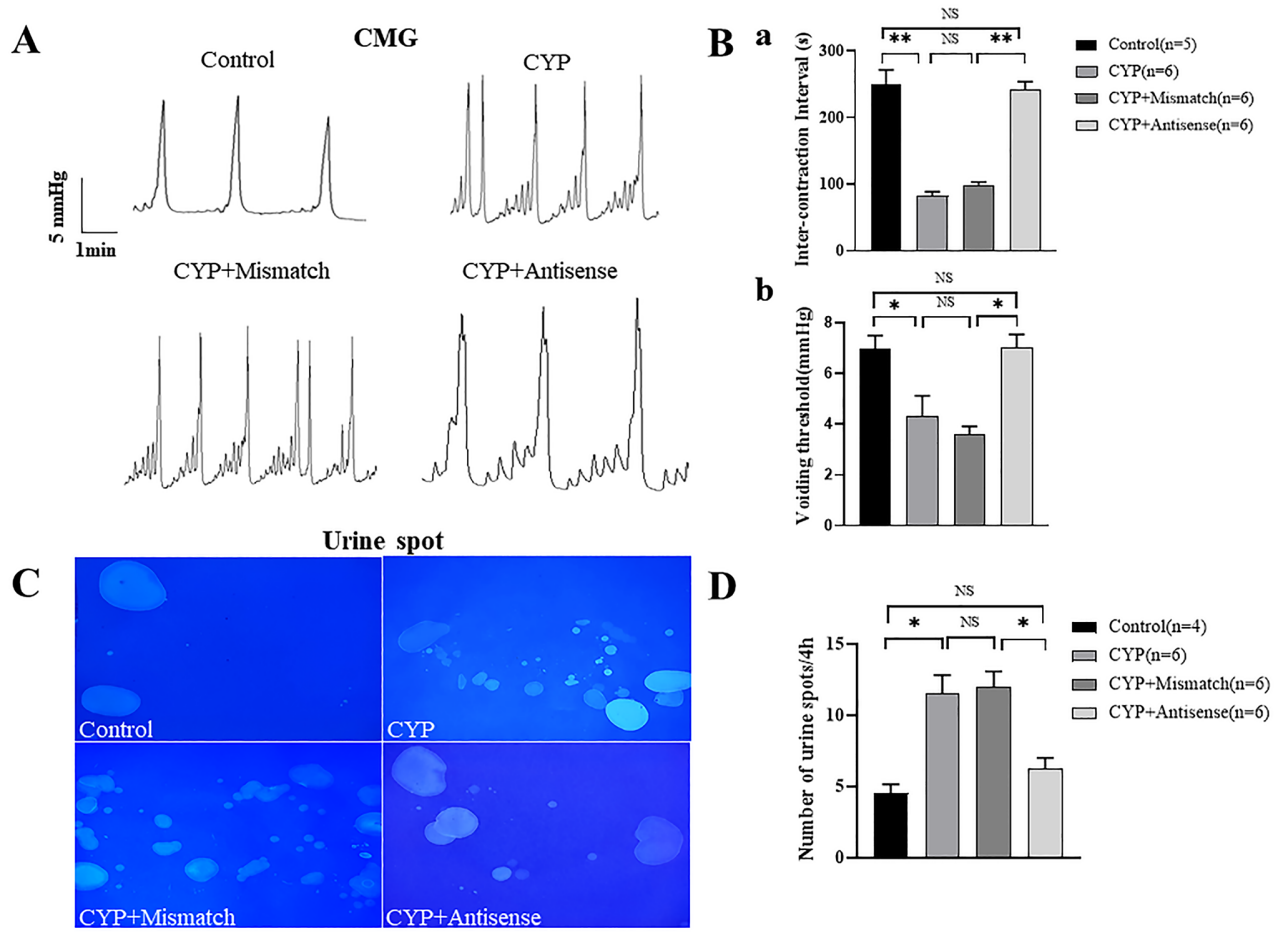
Knockdown Piezo2 expression in sensory neurons reduced bladder overactivity in CYP rats. **(A**) Typical recordings of cystometry measurements under anesthesia in four groups of rats. CYP rats and mismatch + CYP rats exhibit reduced voiding interval and voiding pressure threshold, and knockdown Piezo2 expression (Antisense + CYP) reversed these changes. (**B**) Summary data for voiding interval (Ba) and voiding pressure threshold (Bb) in four groups. *P < 0.05, ** P<0.01. n is the number of rats in each group. **(C**) Typical images of urine spots essay during 4 h in conscious rats from four groups. (**D**) Summary data showing knockdown Piezo2 expression (Antisense + CYP) significantly reduced the urination frequency of CYP rats compared with mismatch + CYP group. *P < 0.05

Bladder overactivity is another presentation in CYP rats. To examine whether increased Piezo2 expression in bladder sensory afferents contributes to bladder overactivity, voiding behaviors were measured under anesthesia and awake conditions. CMG recordings under anesthesia showed that CYP rats and mismatched + CYP rats exhibited decreased inter-contraction intervals and decreased pressure thresholds for voiding compared to the control rats (Fig. 7A and B), suggesting an overactive bladder. Meanwhile, the above changes in CMG parameters were significantly reversed in anti-sense + CYP rats (Fig. 7A and B). The suppressive effects of Piezo2 anti-sense ODN treatment on bladder hyperactivity were confirmed in urine spot experiments in freely moving rats (Fig. 7C and D).

## Discussion

The Piezo2 channel has recently been shown to be involved in mechanical allodynia in inflammation or neuropathic conditions [11, 15, 16]. To our knowledge, this is the first study to examine Piezo2 involvement in IC-associated mechanical allodynia. Rats with CYP-induced chronic cystitis were used as an IC model, and the main findings were that (1) Piezo2 channels are expressed on most of the bladder primary afferents; (2) CYP-induced cystitis is associated with Piezo2 upregulation in bladder afferent neurons at the mRNA, protein, and functional levels; and (3) knockdown of Piezo2 expression in DRG neurons by intrathecal injection of Piezo anti-sense ODNs suppressed mechanical stimulation evoked referred bladder pain and bladder hyperactivity in CYP rats. Our results suggest that upregulation of Piezo2 channels is involved in the development of mechanical allodynia and bladder hyperactivity in CYP-induced cystitis.

One previous study showed that Piezo2-knockout mice exhibited a reduced voiding frequency, and humans lacking functional Piezo2 reported a deficient bladder-filling sensation [32]. Thus, the important role of Piezo2 in sensing the bladder-filling stretch and normal micturition reflex was suggested [32]. However, the authors of this prior study did not investigate the Piezo2 channel expression on bladder sensory afferents [32]. In our study, with FISH and whole-mount bladder nerve staining methods, we found that Piezo2 channels were expressed in most (> 90%) of rat L6–S1 DRG neurons innervating the bladder (Figs. 2A and 3A) and their peripheral nerve terminals in the bladder mucosa (Fig. 2B–E). Piezo2 channels are expressed on both A-type fibers (NF200^+^, Fig. 2E) and pain-associated C-type fibers, which include peptidergic (CGRP^+^), non-peptidergic (IB4^+^), and TRPV1^+^ fibers (Fig. 2B–D). It is generally thought that conscious voiding is dependent on Aδ-fiber bladder afferents, even though both Aδ-fiber and C-fiber bladder afferents are mechanoceptive, whereas C-fiber afferents are responsible for bladder nociceptive responses [33]. Our findings provide the basis for the physiological role of the Piezo2 channel in normal bladder sensory function (either mechanical transduction or nociception) as well as potential roles in pathological conditions such as chronic bladder pain and bladder overactivity. The finding that > 90% of rat L6–S1 DRG neurons expressed Piezo2 (Fig. 2A) is consistent with the pan-expression of Piezo2 in rat L4/L5 DRGs [19] and rat trigeminal ganglion [34] but contrasts with reports of 20–60% expression in mouse DRG neurons [8, 14, 35, 36]. This difference may be attributed to different techniques applied (in situ hybridization vs. immunofluorescence) or may suggest there is a species difference in Piezo2 channel expression.

One of our important findings is that CYP-induced cystitis is associated with the upregulation of Piezo2 expression and function in bladder primary afferents. The exact mechanisms underlying Piezo2 upregulation were not examined further in our study and remain unknown to us at this time. Inflammation mediator bradykinin has been reported to upregulate Piezo2 activity and contribute to mechanical hyperalgesia in inflammation conditions [11]. Another inflammation mediator, NGF, enhanced Piezo2-mediated currents in a subpopulation of peptidergic C-fiber nociceptors and has been suggested contribute to NGF-induced mechanical hyperalgesia during inflammation [37]. NGF has also been shown to be involved in the sensitization of bone afferents to mechanical stimulation [18]. Meanwhile, adenosine triphosphate has been shown to upregulate Piezo2 expression via cAMP/EPAC1 [13] and promote mechanical allodynia after trigeminal nerve compression injury [16]. Increased concentrations of the above inflammation mediators (bradykinin, NGF, and adenosine triphosphate) in the bladder wall or blood plasma have been reported in IC patients [38–42]. Thus, these inflammation mediators may be the underlying mechanism for enhanced activity of Piezo2 in bladder primary sensory afferents. Further study of the regulatory effects of these inflammation mediators on Piezo2 in bladder sensory afferents and the cellular pathways is warranted.

In addition to its critical role in touch and proprioception, Piezo2 has recently been shown to be involved in somatic mechanical allodynia in chronic inflammatory or neuropathic pain conditions [11–16]. In IC patients, pain was usually evoked by bladder filling, suggesting mechanical allodynia. In our study, knockdown of Piezo2 expression in DRG neurons significantly reduced mechanical stimulation–evoked referred bladder pain in CYP rats (Fig. 6), indicating the involvement of Piezo2 in bladder mechanical allodynia. Our finding also suggests that Piezo2 plays similar roles in visceral pain as well as somatic pain. Regarding the mechanisms underlying mechanical allodynia, even though central sensitization may play a major role [43], nociceptor sensitization has been proposed to be the primary driver [44]. In the bladder, both Aδ-fiber and C-fiber sensory afferents are mechanoceptive [29, 45], and their sensitization has been shown to contribute to bladder pain [46]. In our study, Piezo2 upregulation was observed in both small- (C-fiber) and medium-sized (Aδ-fiber) DRG neurons innervating the bladder in CYP rats (Fig. 3). Our findings suggest that Piezo2 channels on both classes of primary afferents (C-fibers and Aδ-fibers) play an important role in IC-associated mechanical allodynia.

Bladder hyperactivity is another presentation of IC. In our study, knockdown of Piezo2 expression in DRG neurons also inhibits bladder hyperactivity in CYP rats (Fig. 7). Given the important role of bladder afferent sensitization in bladder hyperactivity in IC patients [47] and the Piezo2 channel upregulation in bladder sensory afferents found in our study (Figs. 3, 4 and 5), it is not surprising for us that reducing Piezo2 expression also inhibited bladder overactivity. Our finding may also indicate that manipulations targeting Piezo2 may be potential therapeutic approaches for overactive bladder patients.

Our study has several limitations. First, we only investigated the role of the Piezo2 channel in a CYP model, which reflects bladder-centric factors, but not other factors observable in IC patients, such as psychological stress. Second, because Piezo2-knockout rats were not available to us, only a Piezo2-knockdown technique was applied to test the role of Piezo2. Third, we only observed the changes of Piezo2 expression in DRG neurons and did not observe changes in other pain pathways such as the spinal dorsal horn. However, Piezo2 expression in the rat spinal dorsal horn has been previously revealed [19]. Thus, Piezo2 inhibition in the dorsal horn induced by intrathecal Piezo2 anti-sense ODNs may also contribute to the reduction of mechanical allodynia.

In conclusion, our findings provide the first evidence that Piezo2 on bladder sensory afferents contributes to the development of mechanical allodynia in IC conditions. Given the current difficulty in managing IC-related bladder pain, targeting the Piezo2 channel might be an attractive therapeutic approach for IC-related mechanical allodynia. For example, when Piezo2 antagonists are available, their intravesical application might have potential for relieving mechanical allodynia in IC patients.

## Electronic Supplementary Material

Below is the link to the electronic supplementary material.

Supplementary Material 1

## Data Availability

Data will be made available on request.
