# Supplementary figures and images for: Piezo2 Channel Upregulation is Involved in Mechanical Allodynia in CYP-Induced Cystitis Rats
Source: Mol Neurobiol. 2023 May 25;60(9):5000–12. doi: 10.1007/s12035-023-03386-9 (PMC10415424; doi:10.1007/s12035-023-03386-9)

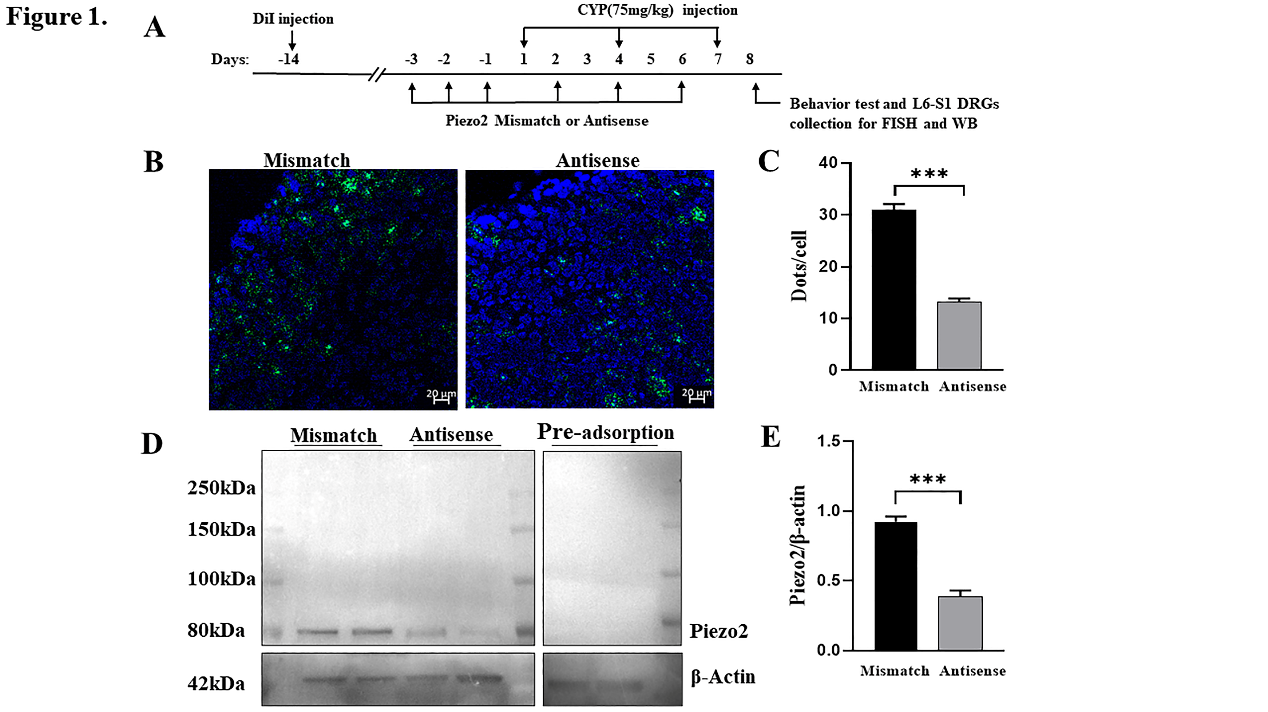


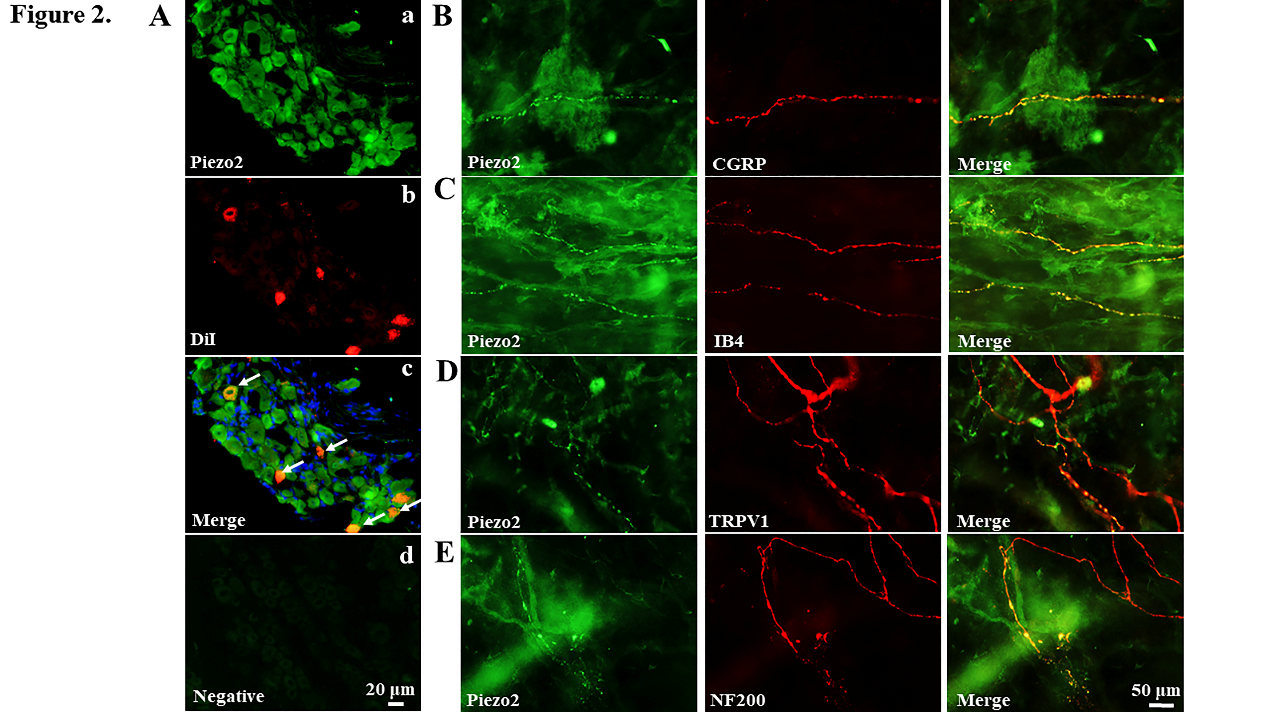


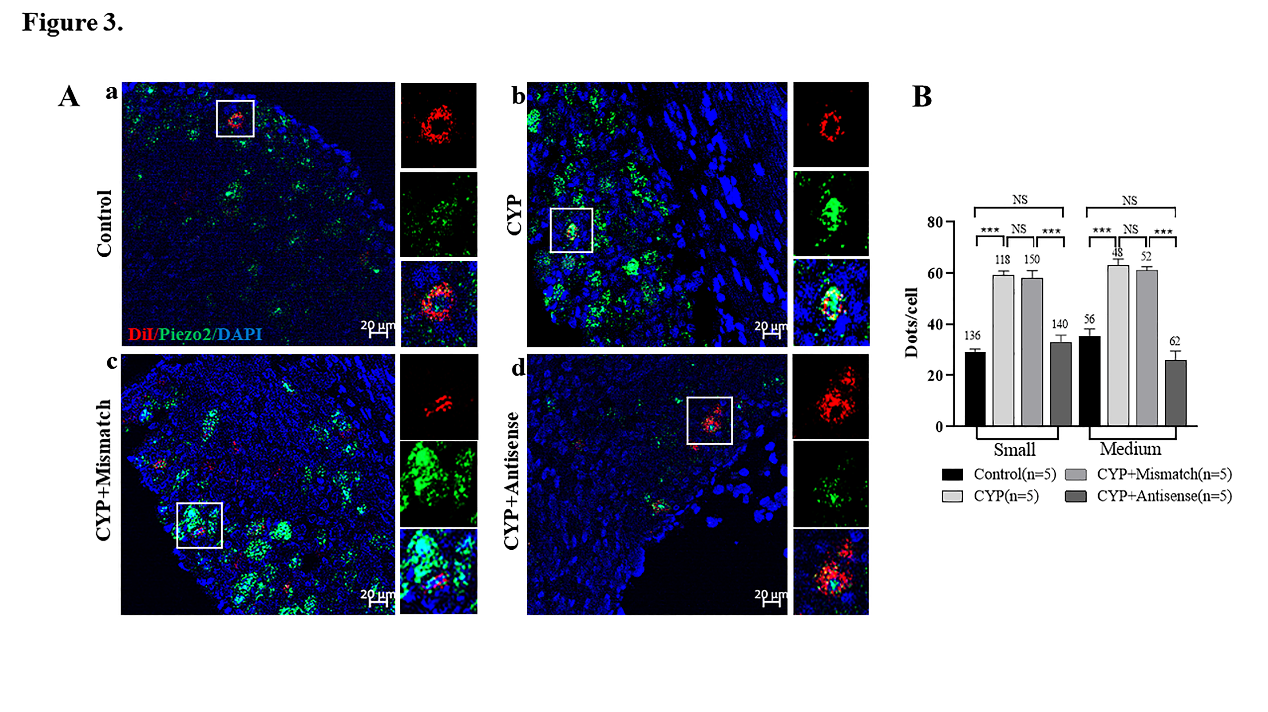


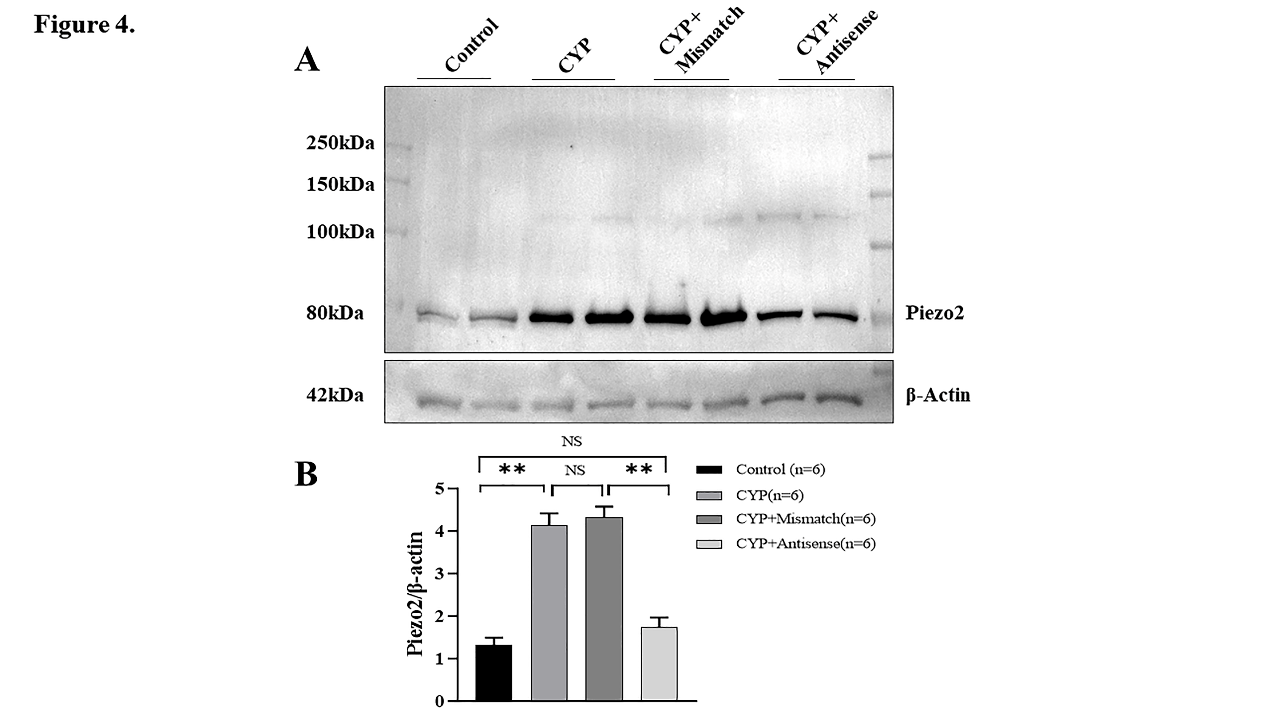


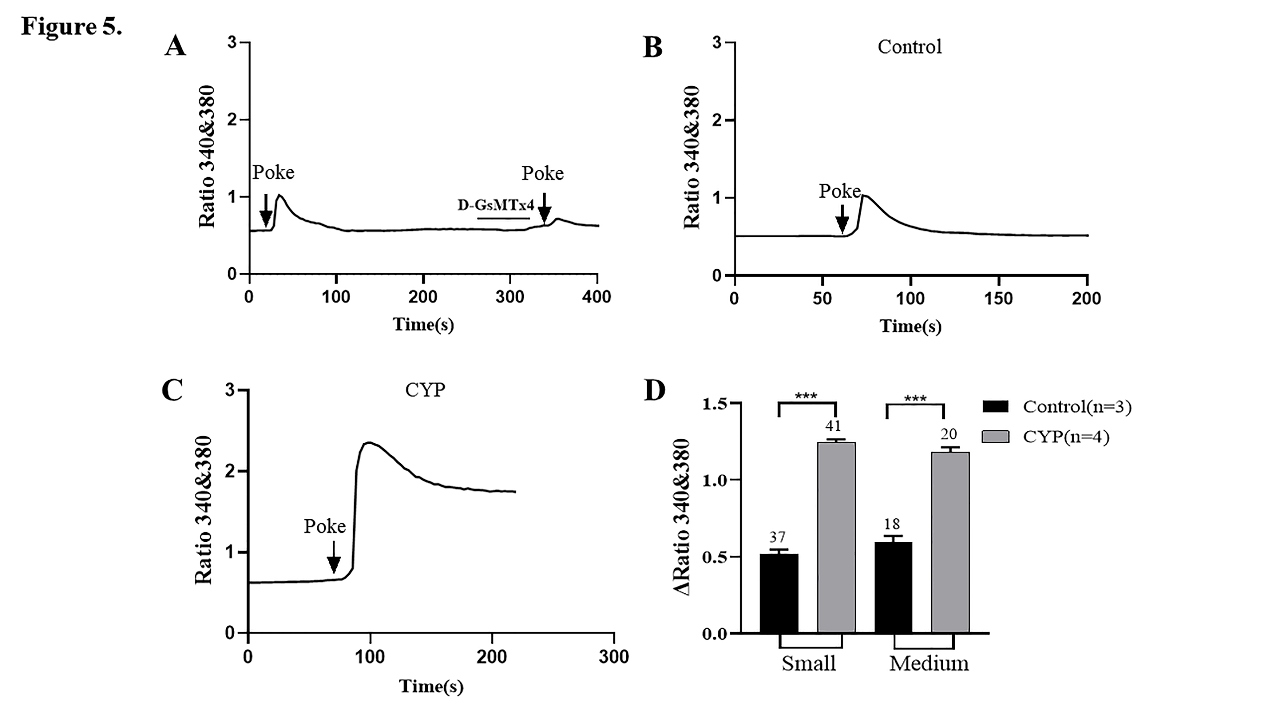


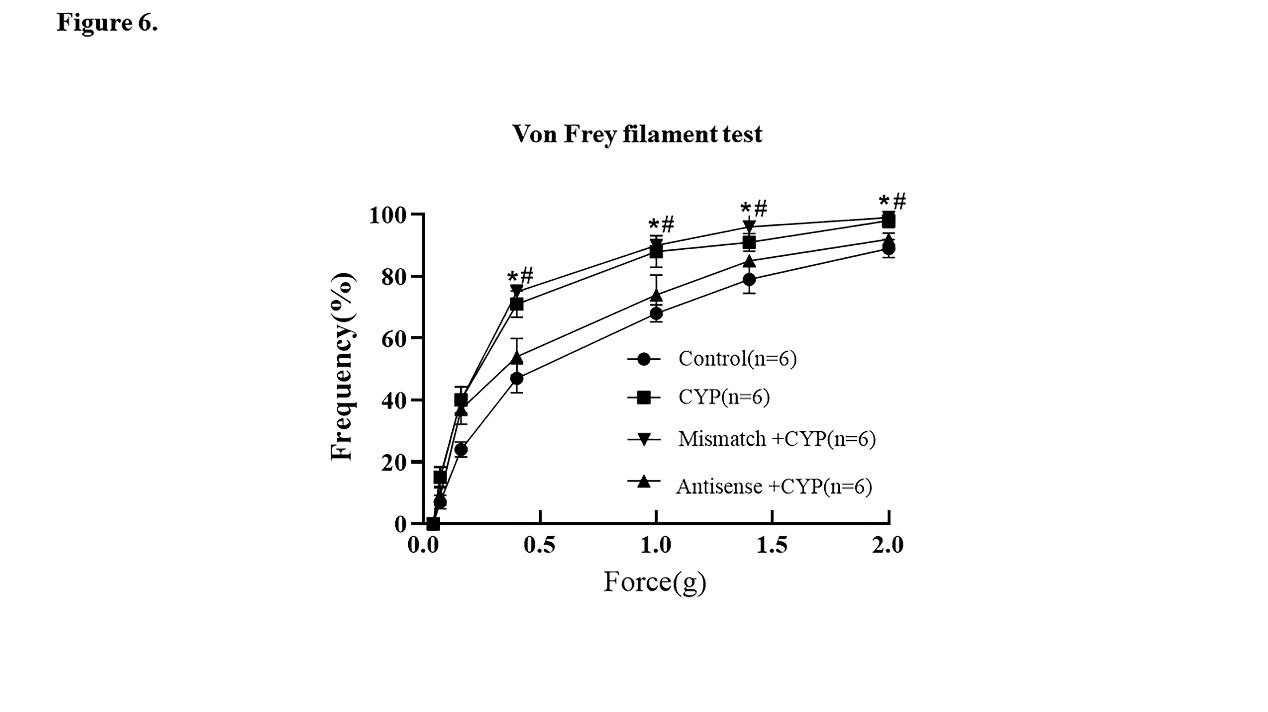


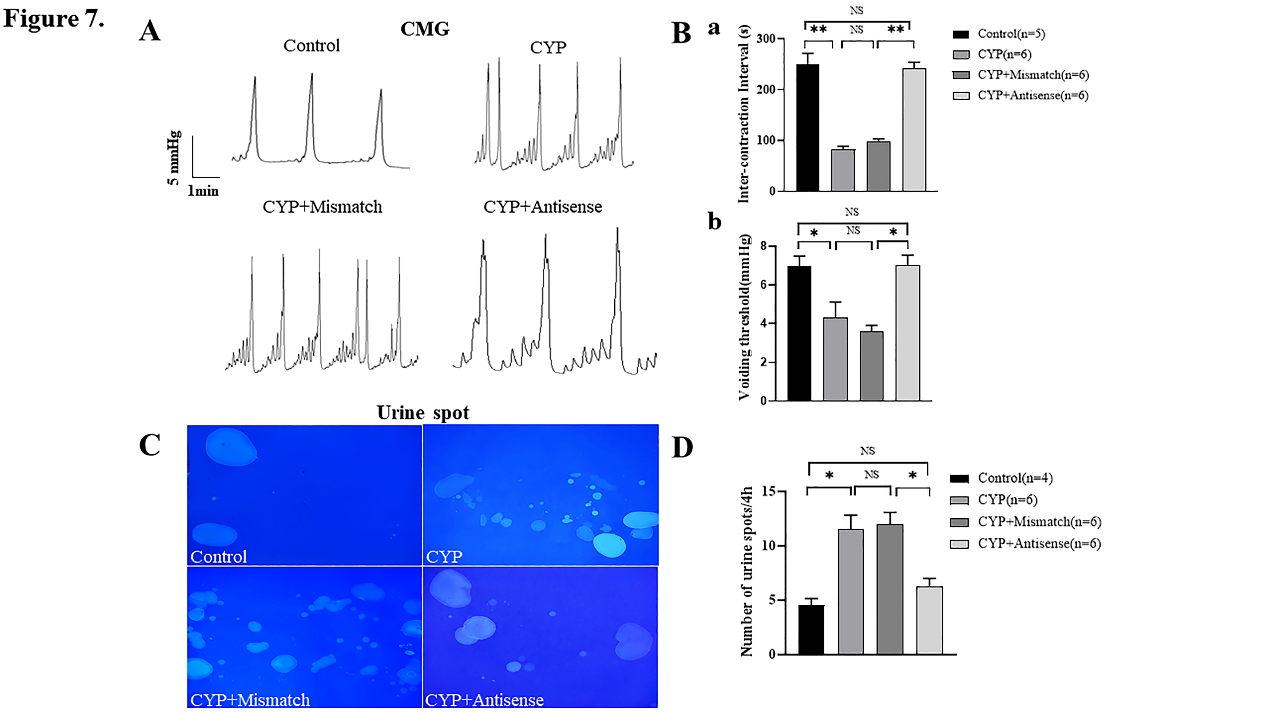

Supplement: Supplementary file 1 — Supplementary Material 1 [file 12035_2023_3386_MOESM1_ESM.docx]
